# NDM-1 Metallo-β-Lactamase and ArmA 16S rRNA methylase producing *Providencia rettgeri* clinical isolates in Nepal

**DOI:** 10.1186/1471-2334-14-56

**Published:** 2014-02-03

**Authors:** Tatsuya Tada, Tohru Miyoshi-Akiyama, Rajan K Dahal, Manoj K Sah, Hiroshi Ohara, Kayo Shimada, Teruo Kirikae, Bharat M Pokhrel

**Affiliations:** 1 Department of Infectious Diseases, Research Institute, National Center for Global Health and Medicine, 1-21-1 Toyama, Shinjuku, Tokyo 162-8655, Japan; 2 Department of International Medical-Cooporation, National Center for Global Health and Medicine, Shinjuku, Tokyo Japan; 3 Department of Microbiology, Institute of Medicine, Tribhuvan University, Maharajgunj, Kathmandu, Nepal

**Keywords:** NDM-1, OXA-72, 16S rRNA methylase, *Providencia rettgeri*, Molecular epidemiology

## Abstract

**Background:**

Drug-resistant *Providencia rettgeri* producing metallo-β-lactamase and 16S rRNA methylase has been reported in several countries. We analyzed *P. rettgeri* clinical isolates with resistance to carbapenems and aminoglycosides in a hospital in Nepal.

**Methods:**

Five clinical isolates of multidrug-resistant *P. rettgeri* were obtained in a hospital in Nepal. Antimicrobial susceptibilities were determined using the microdilution method and entire genomes were sequenced to determine drug-resistant genes. Epidemiological analysis was performed by pulsed-field gel electrophoresis.

**Results:**

Four of the 5 isolates were resistant to carbapenems (imipenem and meropenem), with MICs ≥16 mg/L, with the remaining isolate showing intermediate resistance to imipenem, with an MIC of 2 mg/L and susceptibility to meropenem with an MIC ≤1 mg/L. All 5 isolates had *bla*_VEB-1_. Of the 4 carbapenem-resistant strains, 3 had *bla*_NDM-1_ and 1 had *bla*_OXA-72_. All isolates were highly resistant to aminoglycosides (MICs ≥1,024 mg/L) and harbored *armA*. As the result of pulsed-field gel electrophoresis pattern analysis in the 5 *P. rettgeri* isolates, 4 had identical PFGE patterns and the fifth showed 95.7% similarity.

**Conclusions:**

This is the first report describing multidrug-resistant *P. rettgeri* strains harboring *bla*_NDM-1_ or *bla*_OXA-72_ and *armA* isolated from patients in Nepal.

## Background

*Providencia rettgeri* has been associated with hospital acquired infections, including catheter-related urinary tract infections, bacteremia, skin infections, diarrhea, and gastroenteritis [1,2]. To date, there have been 5 reports of *P. rettgeri* isolates harboring metallo-β-lactamase (MBL) encoding genes, including IMP-type MBL producers in Japan [3,4]; VIM-type MBL, PER-1 extended-spectrum β-lactamase (ESBL) and 16S rRNA methylase ArmA in Korea [5]; and NDM-type MBL in Israel [6] and Brazil [7].

NDM-type MBL was initially identified in *Klebsiella pneumoniae* and *Escherichia coli* in 2009 in Sweden [8]. Since then, NDM-1-producing *Enterobacteriaceae* have been isolated in various parts of the world [9,10].

Exogenously acquired 16S rRNA methylase genes responsible for very high levels of resistance to various aminoglycosides are widely distributed among *Enterobacteriaceae* and glucose-nonfermentative microbes [11]. Gram-negative pathogens producing 16S rRNA methylase ArmA have been isolated in various countries [11].

Although co-production of several resistance determinants is not rare in *Enterobacteriaceae*[12-16], it is less common in *P. rettgeri*[5]. We describe here *P. rettgeri* clinical isolates from Nepal that produce carbapenemase (NDM-1 or OXA-72) and 16S rRNA methylase (ArmA).

## Methods

### Bacterial strains

Five *P. rettgeri* clinical isolates were obtained from May to July 2012 from 5 patients at Tribhuvan University Teaching Hospital in Kathmandu, Nepal. Three isolates were from sputum and 2 from pus at surgical sites. Samples were obtained as part of standard patient care. Phenotypical identification [17] was confirmed by API 32GN (BioMérieux, Mercy l'Etoile, France) and 16S rRNA sequencing (1,497 bp) [18,19].

### Antimicrobial susceptibilities

MICs were determined using the microdilution method, according to the guidelines of the Clinical Laboratory Standards Institute (CLSI) [20]. Breakpoints to antibiotics were determined. The modified Hodge test, the meropenem-sodium mercaptoacetic acid double-disk synergy test (Eiken Chemical, Tokyo, Japan) and E-test (imipenem/EDTA) (AB Biodisk, Solna, Sweden) were performed.

### Entire genome sequencing

The entire genomes of these isolates were extracted and sequenced by MiSeq (Illumina, San Diego, CA). CLC genomics workbench version 5.5 (CLC bio, Tokyo, Japan) was used for de novo assembly of reads and to search for 923 drug-resistance genes, including genes encoding β-lactamases, 16S rRNA methylases and aminoglycoside-acethyl/adenylyltransferases; point mutations in the *gyrA*, *parC* and *pmrCAB* operons; and point mutations in the *fos* genes, including *fosA*, *fosA2*, *fosA3*, *fosC* and *fosC2*.

### Pulsed-field gel electrophoresis (PFGE) and southern hybridization

PFGE analysis was performed as described [3]. An 813 bp probe for *bla*_NDM-1_ was synthesized by PCR amplification using the primers 5′-atggaattgcccaatattatgcac-3′ (forward) and 5′-tcagcgcagcttgtcggccatgcggg-3′ (reverse), and a 780 bp probe for *bla*_OXA-72_ was synthesized using the primers 5′-agtttctctcagtgcatgttcatctat-3′ (forward) and 5′-agaaccagacattccttctttcatttc-3′ (reverse). Southern hybridization to detect *bla*_NDM-1_ and *bla*_OXA-72_ was performed using these probes, which were detected using DIG High Prime DNA labeling and detection starter kit II (Roche Diagnostics, Mannheim, Germany).

### Nucleotide sequence accession numbers

The nucleotide sequences surrounding *bla*_NDM-1_ and *bla*_OXA-72_ have been deposited in GenBank with the accession number AB828598 and AB857844, respectively.

### Ethical approval

The study protocol was reviewed and approved by the Institutional Review Board of the Institute of Medicine, Tribhuvan University (ref. 6-11-E) and the Biosafety Committee, National Center for Global Health and Medicine (approval number: 23-M-49).

## Results

### Antimicrobial susceptibilities

Four of the 5 isolates were resistant to carbapenems (doripenem, imipenem and meropenem) and piperacillin/tazobactam, whereas the fifth was susceptible to piperacillin/tazobactam, doripenem and meropenem and showed intermediate resistance to imipenem (Table 1). All 5 isolates were highly resistant to cephalosporins (ceftazidime and cefepime), aztreonam, aminoglycosides (arbekacin, amikacin and gentamicin), ciprofloxacin, colistin and fosfomycin, and all 5 showed intermediate resistance to tigecycline. The four isolates resistant to carbapenems were negative with the modified Hodge test, but three of the four isolates were positive with the meropenem-sodium mercaptoacetic acid double-disk synergy test and E-test/EDTA.

**Table 1:** **Summary of the characteristics of the 5 *P. rettegeri *** **strains, including antimicrobial resistance profiles and resistant genes**

**Strains**	**Tissue sources**	**Infection**		**MIC (mg/L)**														**Antibiotics resistant genes**
			**PIP**	**TZP**	**CAZ**	**FEP**	**IPM**	**DPM**	**MEM**	**ATM**	**ABK**	**AMK**	**GEN**	**CIP**	**CST**	**FOF**	**TIG**	
IOMTU1	Pus	SSI	1,024	512	>1,024	64	32	16	64	1,024	>1,024	>1,024	>1,024	128	>128	512	4	*bla*_NDM-1_, *bla*_OXA-10_, *bla*_VEB-1_, *bla*_TEM-1_, *bla*_ADC-67_, *armA*, *aadA1*, *aadA2*
IOMTU4	Sputum	NLRTI	1,024	128	>1,024	256	16	16	32	1,024	>1,024	>1,024	>1,024	>256	>128	512	4	*bla*_OXA-72_, *bla*_OXA-10_, *bla*_VEB-1_, *bla*_TEM-1_, *bla*_ADC-67_, *armA*, *aadA1*
IOMTU91	Sputum	NLRTI	>1,024	1,024	>1,024	1,024	64	32	64	1,024	>1,024	>1,024	>1,024	256	128	128	4	*bla*_NDM-1_, *bla*_OXA-10_, *bla*_VEB-1_, *bla*_TEM-1_, *bla*_ADC-67_, *armA*, *aadA1*
IOMTU94	Pus	SSI	1,024	4	>1,024	256	2	1	1	>1,024	1,024	1,024	>1,024	256	>128	1,024	4	*bla*_OXA-10_, *bla*_VEB-1_, *bla*_TEM-1_, *bla*_ADC-67_, *armA*, *aadA1*
IOMTU99	Sputum	NLRTI	>1,024	512	>1,024	128	64	32	64	1,024	>1,024	>1,024	>1,024	>256	>128	1,024	4	*bla*_NDM-1_, *bla*_VEB-1_, *bla*_OXA-10_, *bla*_TEM-1_, *bla*_ADC-67_, *armA*, *aadA1*

SSI, surgical site infection; NLRTI, nosocomial lower respiratory tract infection PIP, piperacillin; TZP, piperacillin/tazobactam; CAZ, ceftazidime; FEP, cefepime; IPM, imipenem; DPM, doripenem; MEM, meropenem; ATM, aztreonam; ABK, arbekacin; AMK, amikacin; GEN, gentamicin; CIP, ciprofloxacin; CST, colistin; FOF, fosfomycin; TIG, tigecycline.

### Drug-resistant genes

All 5 isolates tested had several genes associated with β-lactam and aminoglycoside-resistance (Table 1). These isolates had *bla*_VEB-1_, *bla*_OXA-10_, *bla*_TEM-1_, *bla*_ADC-67_ (*ampC*), *armA* and *aadA1*; 3 had *bla*_NDM-1_; and 1 had *bla*_OXA-72_. None of these isolates had any other β-lactamase encoding genes, including the class A genes *bla*_SHVs_ and *bla*_CTX-Ms_; the class B genes *bla*_AIM_, *bla*_DIM_, *bla*_FIM_, *bla*_GIM_, *bla*_IMPs_, *bla*_INDs_, *bla*_KHM_, *bla*_SIM_, *bla*_SMB_, *bla*_SPM_, *bla*_TMBs_, and *bla*_VIMs_; or the class D gene *bla*_OXAs_ except for *bla*_OXA-10_ and *bla*_OXA-72_. None had other genes encoding 16S rRNA methylases or aminoglycoside acetyl/adenylyltransferases. All 5 isolates had point mutations in the quinolone-resistance-determining regions of *gyrA* and *parC*, with amino acid substitutions of S83I and D87E in GyrA and S80I in ParC, but none had any mutations in the *pmrCAB* operon and *fos* genes. All sequences of the drug-resistant genes tested were identical to those registered in GenBank.

### PFGE and southern hybridyzation

**Figure 1:**
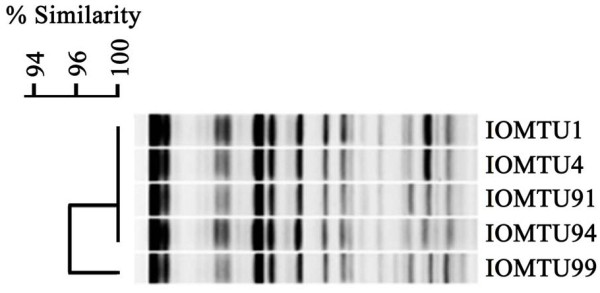
**PFGE profiles obtained following *Sfi*** **I digestion of *P. rettgeri *** **chromosomes.**

Of the 5 *P. rettgeri* isolates, 4 had identical PFGE patterns and the fifth showed 95.7% similarity (Figure 1). Three of these isolates had a plasmid harboring *bla*_NDM-1_ and one had a plasmid harboring *bla*_OXA-72_, with plasmid sizes ranging from 9.42 to 23.1 kbp (data not shown).

### Genomic structures surrounding *bla*_NDM-1_ and *bla*_OXA-72_

The genetic environments surrounding *bla*_NDM-1_ (Accession no. AB828598) was *bla*_NDM-1_*-ble*_MBL_*-trpF-dsbC-cutA1*. All 3 isolates harboring *bla*_NDM-1_ (IOMTU1, 91 and 99) had the same genetic environments. The *bla*_OXA-72_ gene was flanked by conserved inverted repeats at the XerC/XerD binding sites [21], indicating mobilization by site-specific recombination mechanisms. The *rep1* gene was located downstream of *bla*_OXA-72_ (Accession no. AB857844).

## Discussion

The relatively high MICs to piperacillin/tazobactam and carbapenems of the five *P. rettgeri* isolates were likely due to the presence of *bla*_NDM-1_ or *bla*_OXA-72_. The enzymatic activities of metallo-β-lactamases, including NDM-1, were not inhibited by tazobactam [22], a β-lactamase inhibitor, in agreement with the MIC profiles of these isolates to piperacillin/tazobactam. The high MICs of all 5 isolates to ceftazidime, cefepime and aztreonam were likely due to the presence of *bla*_VEB-1_[23], and the presence of *armA* in these isolates was likely associated with their extremely high resistance to all aminoglycosides tested [11]. Point mutations in the quinolone-resistance-determining regions of *gyrA* and *parC* have been associated with high resistance to quinolones [24]. Point mutations in *pmrCAB* operon have been associated with the resistance of *Acinetobacter* spp. [25] and *Pseudomonas aeruginosa*[26] to polymxyin and colistin; and the presence of *fos* genes, including *fosA*, *fosA2*, *fosA3*, *fosC* and *fosC2*, has been associated with resistance to fosfomycin in Gram-negative bacteria [27-29].

Plasmids containing *bla*_NDM-1_ or *bla*_OXA-72_ may be disseminated among Gram-negative pathogens in Nepal. The genetic environments surrounding *bla*_NDM-1_ in our *P. rettgeri* strains (*bla*_NDM-1_-*ble*_MBL_-*trpF*-*dsbC*-*cutA1*) were also observed in other plasmids, including *A. baumannii* plasmid pAbNDM-1 from China (Accession no. JN377410), *Citrobacter freundii* plasmid pYE315203 from China (Accession no. JX254913), *E. coli* plasmid pNDM102337 from Canada (Accession no. JF714412), *K. pneumoniae* plasmid pKP-NCGM18-1 from Nepal (Accession no. AB824738) [30], *K. pneumoniae* plasmids pKPX-1, pKPN5047 and pNDM-HN380 from China (Accession nos. AP012055, KC311431 and JX104760, respectively), and *P. rettgeri* plasmid pFR90 (Accession no. JQ362415) from China. In addition, the genetic structures of OXA-72 producing *Acinetobacter* spp [31-34] and *K. pneumoniae* (Accession no. JX268653 and AB825955 deposited in 2012 and 2013, respectively) had the same genetic structure (*bla*_OXA-72_-*rep1*) as our strain of *P. rettgeri*.

## Conclusions

To our knowledge, this is the first report describing *P. rettgeri* strains harboring *bla*_NDM-1_ or *bla*_OXA-72_ and *armA* isolated from patients in Nepal. These 5 strains were highly resistant to both β-lactams and aminoglycosides and expanded in a clonal manner in the hospital.

## Competing interests

The authors declare that they have no competing interest.

## Authors’ contributions

TT: Performed PCR and sequencing, analyzed data and drafted the manuscript. TMA: Performed entire genome sequencing. RKD and MKS: Performed drug susceptibility tests. HO: Supervised this study. KS: Performed pulsed-field gel electrophoresis and its pattern analysis. TK and BMP: Designed protocols and supervised this study. All authors read and approved the final manuscript.

## Pre-publication history

The pre-publication history for this paper can be accessed here:

http://www.biomedcentral.com/1471-2334/14/56/prepub

## References

[B1] Stock I Wiedemann B Natural antibiotic susceptibility of *Providencia stuartii*, *P. rettgeri*, *P. alcalifaciens* and *P. rustigianii* strains J Med Microbiol 1998 14 629 642 10.1099/00222615-47-7-629 9839568

[B2] Yoh M Matsuyama J Ohnishi M Takagi K Miyagi H Mori K Park KS Ono T Honda T Importance of *Providencia* species as a major cause of travellers' diarrhoea J Med Microbiol 2005 14 1077 1082 10.1099/jmm.0.45846-0 16192440

[B3] Shiroto K Ishii Y Kimura S Alba J Watanabe K Matsushima Y Yamaguchi K Metallo-beta-lactamase IMP-1 in *Providencia rettgeri* from two different hospitals in Japan J Med Microbiol 2005 14 1065 1070 10.1099/jmm.0.46194-0 16192438

[B4] Nishio H Komatsu M Shibata N Shimakawa K Sueyoshi N Ura T Satoh K Toyokawa M Nakamura T Wada Y Orita T Kofuku T Yamasaki K Sakamoto M Kinoshita S Aihara M Arakawa Y Metallo-beta-lactamase-producing Gram-negative bacilli: laboratory-based surveillance in cooperation with 13 clinical laboratories in the Kinki region of Japan J Clin Microbiol 2004 14 5256 5263 10.1128/JCM.42.11.5256-5263.2004 15528723 PMC525181

[B5] Lee HW Kang HY Shin KS Kim J Multidrug-resistant *Providencia* isolates carrying *bla*_PER-1_, *bla*_VIM-2_, and *armA* J Microbiol 2007 14 272 274 17618235

[B6] Gefen-Halevi S Hindiyeh MY Ben-David D Smollan G Gal-Mor O Azar R Castanheira M Belausov N Rahav G Tal I Mendelson E Keller N Isolation of genetically not related *bla*_NDM-1_ positive *Providencia rettgeri* in Israel J Clin Microbiol 2013 14 1642 1643 10.1128/JCM.00381-13 23486709 PMC3647926

[B7] Carvalho-Assef AP Pereira PS Albano RM Beriao GC Chagas TP Timm LN Da Silva RC Falci DR Asensi MD Isolation of NDM-producing *Providencia rettgeri* in Brazil J Antimicrob Chemotherin press 10.1093/jac/dkt298 23869051

[B8] Yong D Toleman MA Giske CG Cho HS Sundman K Lee K Walsh TR Characterization of a new metallo-beta-lactamase gene, *bla*_NDM-1_, and a novel erythromycin esterase gene carried on a unique genetic structure in *Klebsiella pneumoniae* sequence type 14 from India Antimicrob Agents Chemother 2009 14 5046 5054 10.1128/AAC.00774-09 19770275 PMC2786356

[B9] Cornaglia G Giamarellou H Rossolini GM Metallo-beta-lactamases: a last frontier for beta-lactams? Lancet Infect Dis 2011 14 381 393 10.1016/S1473-3099(11)70056-1 21530894

[B10] Pillai DR McGeer A Low DE New Delhi metallo-beta-lactamase-1 in Enterobacteriaceae: emerging resistance CMAJ 2011 14 59 64 10.1503/cmaj.101487 21220461 PMC3017254

[B11] Wachino J Arakawa Y Exogenously acquired 16S rRNA methyltransferases found in aminoglycoside-resistant pathogenic Gram-negative bacteria: an update Drug Resist Updat 2012 14 133 148 10.1016/j.drup.2012.05.001 22673098

[B12] Sheng WH Badal RE Hsueh PR SMART Program Distribution of extended-spectrum beta-Lactamases, AmpC beta-Lactamases, and carbapenemases among enterobacteriaceae isolates causing intra-abdominal infections in the Asia-Pacific region: results of the Study for Monitoring Antimicrobial Resistance Trends (SMART) Antimicrob Agents Chemother 2013 14 2981 2988 10.1128/AAC.00971-12 23587958 PMC3697370

[B13] Bueno MF Francisco GR O'Hara JA de Oliveira Garcia D Doi Y Co-production of 16S Ribosomal RNA Methyltransferase RmtD and RmtG with KPC-2 and CTX-M-group ESBLs in *Klebsiella pneumoniae* Antimicrob Agents Chemother 2013 14 2397 2400 10.1128/AAC.02108-12 23459483 PMC3632927

[B14] Galani I Souli M Panagea T Poulakou G Kanellakopoulou K Giamarellou H Prevalence of 16S rRNA methylase genes in Enterobacteriaceae isolates from a Greek university hospital Clin Microbiol Infect 2012 14 E52 E54 10.1111/j.1469-0691.2011.03738.x 22264302

[B15] Zacharczuk K Piekarska K Szych J Zawidzka E Sulikowska A Wardak S Jagielski M Gierczynski R Emergence of *Klebsiella pneumoniae* coproducing KPC-2 and 16S rRNA methylase ArmA in Poland Antimicrob Agents Chemother 2011 14 443 446 10.1128/AAC.00386-10 20956599 PMC3019640

[B16] Wu Q Liu Q Han L Sun J Ni Y Plasmid-mediated carbapenem-hydrolyzing enzyme KPC-2 and ArmA 16S rRNA methylase conferring high-level aminoglycoside resistance in carbapenem-resistant *Enterobacter cloacae* in China Diagn Microbiol Infect Dis 2010 14 326 328 10.1016/j.diagmicrobio.2009.10.003 19903584

[B17] Tang YW Ellis NM Hopkins MK Smith DH Dodge DE Persing DH Comparison of phenotypic and genotypic techniques for identification of unusual aerobic pathogenic gram-negative bacilli J Clin Microbiol 1998 14 3674 3679 9817894 10.1128/jcm.36.12.3674-3679.1998 PMC105261

[B18] Marchesi JR Sato T Weightman AJ Martin TA Fry JC Hiom SJ Dymock D Wade WG Design and evaluation of useful bacterium-specific PCR primers that amplify genes coding for bacterial 16S rRNA Appl Environ Microbiol 1998 14 795 799 9464425 10.1128/aem.64.2.795-799.1998 PMC106123

[B19] Simmon KE Croft AC Petti CA Application of SmartGene IDNS software to partial 16S rRNA gene sequences for a diverse group of bacteria in a clinical laboratory J Clin Microbiol 2006 14 4400 4406 10.1128/JCM.01364-06 17050811 PMC1698390

[B20] National Committee for Clinical Laboratory Standards Methods for dilution antimicrobial susceptibility tests for bacteria that grow aerobically, 9th ed Approved standard M07-A9 2012 8 Wayne, Pa: Clinical and Laboratory Standards Institute

[B21] D'Andrea MM Giani T D'Arezzo S Capone A Petrosillo N Visca P Luzzaro F Rossolini GM Characterization of pABVA01, a plasmid encoding the OXA-24 carbapenemase from Italian isolates of *Acinetobacter baumannii* Antimicrob Agents Chemother 2009 14 3528 3533 10.1128/AAC.00178-09 19487447 PMC2715606

[B22] Bush K Jacoby GA Updated functional classification of beta-lactamases Antimicrob Agents Chemother 2010 14 969 976 10.1128/AAC.01009-09 19995920 PMC2825993

[B23] Poirel L Naas T Guibert M Chaibi EB Labia R Nordmann P Molecular and biochemical characterization of VEB-1, a novel class A extended-spectrum beta-lactamase encoded by an *Escherichia coli* integron gene Antimicrob Agents Chemother 1999 14 573 581 10049269 10.1128/aac.43.3.573 PMC89162

[B24] Jacoby GA Mechanisms of resistance to quinolones Clin Infect Dis 2005 14 Suppl 2 S120 S126 15942878 10.1086/428052

[B25] Adams MD Nickel GC Bajaksouzian S Lavender H Murthy AR Jacobs MR Bonomo RA Resistance to colistin in *Acinetobacter baumannii* associated with mutations in the PmrAB two-component system Antimicrob Agents Chemother 2009 14 3628 3634 10.1128/AAC.00284-09 19528270 PMC2737849

[B26] Moskowitz SM Ernst RK Miller SI PmrAB, a two-component regulatory system of *Pseudomonas aeruginosa* that modulates resistance to cationic antimicrobial peptides and addition of aminoarabinose to lipid A J Bacteriol 2004 14 575 579 10.1128/JB.186.2.575-579.2004 14702327 PMC305751

[B27] Beharry Z Palzkill T Functional analysis of active site residues of the fosfomycin resistance enzyme FosA from *Pseudomonas aeruginosa* J Biol Chem 2005 14 17786 17791 10.1074/jbc.M501052200 15741169

[B28] Xu H Miao V Kwong W Xia R Davies J Identification of a novel fosfomycin resistance gene (fosA2) in *Enterobacter cloacae* from the Salmon River, Canada Lett Appl Microbiol 2011 14 427 429 10.1111/j.1472-765X.2011.03016.x 21392044

[B29] Wachino J Yamane K Suzuki S Kimura K Arakawa Y Prevalence of fosfomycin resistance among CTX-M-producing *Escherichia coli* clinical isolates in Japan and identification of novel plasmid-mediated fosfomycin-modifying enzymes Antimicrob Agents Chemother 2010 14 3061 3064 10.1128/AAC.01834-09 20404116 PMC2897269

[B30] Tada T Miyoshi-Akiyama T Dahal RK Mishra SK Ohara H Shimada K Kirikae T Pokhrel BM Dissemination of multidrug-resistant *Klebsiella pneumoniae* clinical isolates with various combinations of carbapenemases (NDM-1 and OXA-72) and 16S rRNA methylases (ArmA, RmtC and RmtF) in Nepal Int J Antimicrob Agents 2013 14 372 374 10.1016/j.ijantimicag.2013.06.014 23978353

[B31] Werneck JS Picao RC Carvalhaes CG Cardoso JP Gales AC OXA-72-producing *Acinetobacter baumannii* in Brazil: a case report J Antimicrob Chemother 2011 14 452 454 10.1093/jac/dkq462 21131320

[B32] Wang H Guo P Sun H Wang H Yang Q Chen M Xu Y Zhu Y Molecular epidemiology of clinical isolates of carbapenem-resistant *Acinetobacter* spp. from Chinese hospitals Antimicrob Agents Chemother 2007 14 4022 4028 10.1128/AAC.01259-06 17846127 PMC2151426

[B33] Montealegre MC Maya JJ Correa A Espinal P Mojica MF Ruiz SJ Rosso F Vila J Quinn JP Villegas MV First identification of OXA-72 carbapenemase from *Acinetobacter pittii* in Colombia Antimicrob Agents Chemother 2012 14 3996 3998 10.1128/AAC.05628-11 22508295 PMC3393383

[B34] Goic-Barisic I Towner KJ Kovacic A Sisko-Kraljevic K Tonkic M Novak A Punda-Polic V Outbreak in Croatia caused by a new carbapenem-resistant clone of *Acinetobacter baumannii* producing OXA-72 carbapenemase J Hosp Infect 2011 14 368 369 10.1016/j.jhin.2010.12.003 21316806

